# Pulmonary Valve Replacement in Repaired Tetralogy of Fallot: Midterm Impact on Biventricular Response and Adverse Clinical Outcomes

**DOI:** 10.3389/fped.2022.864455

**Published:** 2022-05-06

**Authors:** Fengpu He, Zicong Feng, Jianhui Yuan, Kai Ma, Keming Yang, Minjie Lu, Sen Zhang, Shoujun Li

**Affiliations:** ^1^Department of Cardiovascular Surgery, The First Affiliated Hospital, Zhejiang University College of Medicine, Hangzhou, China; ^2^Paediatric Cardiac Surgery Centre, National Centre for Cardiovascular Diseases, Peking Union Medical College, Fuwai Hospital, Chinese Academy of Medical Sciences, Beijing, China; ^3^Department of Magnetic Resonance Imaging, National Centre for Cardiovascular Diseases, Peking Union Medical College, Chinese Academy of Medical Sciences, Beijing, China

**Keywords:** pulmonary valve replacement, repaired tetralogy of fallot, pulmonary regurgitation, cardiovascular magnetic resonance, right ventricular reverse remodeling

## Abstract

**Background:**

Pulmonary regurgitation (PR), though well tolerated for short term in patients with repaired tetralogy of Fallot (rTOF), could lead to right ventricular (RV) dysfunction, arrhythmias, and sudden cardiac death. Pulmonary valve replacement (PVR), considered as the gold-standard treatment for PR, is performed to mitigate these late effects. In this study, we aimed to evaluate the midterm outcomes and predictors of adverse clinical outcomes (ACO).

**Methods:**

From May 2014 to December 2017, 42 patients with rTOF undergoing surgical or transcatheter PVR in our department were retrospectively included. Cardiovascular magnetic resonance was performed before PVR (pre-PVR), early after PVR (early post-PVR), and midterm after PVR (midterm post-PVR). Medical history and individual data were collected from medical records. ACO included all-cause death, new-onset arrhythmia, prosthetic valve failure, and repeat PVR.

**Results:**

The median follow-up duration was 4.7 years. PVR was performed at a median age of 21.6 years. There was no early or late death. Freedom from ACO at 3 and 5 years was 88.1 ± 5% and 58.2 ± 9%, respectively. RV end-diastolic volume index (RVEDVI) and end-systolic volume index (RVESVI) significantly reduced early after PVR and further decreased by midterm follow-up (pre-PVR vs. early post-PVR vs. midterm post-PVR: RVEDVI, 155.2 ± 34.7 vs. 103.8 ± 31.2 vs. 95.1 ± 28.6 ml/m^2^, *p* < 0.001; RVESVI, 102.9 ± 28.5 vs. 65.4 ± 28.2 vs. 57.7 ± 23.4 ml/m^2^, *p* < 0.001). Multivariable analysis revealed that the occurrence of ACO was significantly increased in patients with lower left ventricular end-systolic volume index.

**Conclusions:**

A significant reduction of RV volume occurred early after PVR, followed by a further improvement of biventricular function by midterm follow-up. The midterm freedom from ACO was favorable.

## Introduction

Pulmonary regurgitation (PR), largely attributed to the classic surgical repair with the use of a transannular patch, is generally considered well-tolerated in patients with repaired Tetralogy of Fallot (rTOF) for the short term (1). This ongoing valve insufficiency, however, frequently leads to progressive right ventricular (RV) enlargement, adverse clinical outcomes (ACO), and even sudden cardiac death (2–5). As the gold-standard treatment for PR to eliminate these late effects, pulmonary valve replacement (PVR) has been already proven to be associated with reversible RV remodeling, RV normalization, and notable symptomatic benefits (6–8). Nevertheless, many current studies placed great emphasis on the optimal timing and indications for PVR in patients with rTOF. The prior results reporting the improvement of RV function in response to PVR are conflicting (6, 9–12). It is unknown whether the reverse RV remodeling and normalization after PVR will present an ongoing improvement over time, or simply will stabilize after the reduction of RV volume load (13, 14). Following the favorable outcomes previously published by our prospective case-control study (15), this cohort continued to evaluate the midterm results of PVR and investigate potential risk factors for ACO.

## Materials and Methods

### Study Design and Patients Inclusion

This retrospective single-center study complied with the Declaration of Helsinki and was approved by the Ethics Committee of Fuwai Hospital. All patients were provided with written informed consent for examination protocol and medical record review. For the initial inclusion in the study, patients had to fulfill the following criteria: (1) rTOF; (2) PVR performed in our hospital between May 1, 2014, and December 31, 2017; (3) the latest post-PVR cardiovascular magnetic resonance (CMR) performed no more than 5 years following PVR, and no contraindications to CMR; (4) follow-up ≥3 years. CMR was performed at 3 time points: pre-PVR, early post-PVR (minimum, 6 months), and midterm post-PVR (minimum, 36 months) during the entire follow-up. Only patients with a complete CMR imaging data set at all three assessment points were incorporated and analyzed. Of the 45 subjects screened for enrollment, 42 patients met the inclusion criteria described above and formed the study cohort. Demographic and surgical characteristics before exclusion are listed in Supplementary Tables. Medical history and individual data were collected from medical records. Clinical status was obtained through outpatient visits or telephone follow-up with patients or family members, as appropriate. CMR was performed on a 1.5 Tesla magnetic resonance scanner (Magnetom Avanto; Siemens Medical Solutions, Erlangen, Germany). Our protocols for image acquisition and analysis in patients with rTOF have been previously reported (15). The CMR data were analyzed using commercially available software packages (Philips Intellispace Portal).

### PVR Strategy

In the current study, those same indications for asymptomatic patient's referral to surgical or transcatheter PVR were moderate or severe PR with one of the following: (1) right ventricular end-diastolic volume index (RVEDVI) ≥150 ml/m^2^, or (2) right ventricular end-systolic volume index (RVESVI) ≥120 ml/m^2^, or (3) right ventricular ejection fraction (RVEF) <47%. Favorable anatomy and patient's weight, however, need to be considered for transcatheter PVR: (1) pulmonary valve annulus ≤ 30 mm by cardiac computerized tomography, (2) no significant right ventricular outflow tract or main pulmonary artery narrowing, (3) no significant obstruction of the proximal branches of pulmonary artery, and (4) patent central veins (16).

### Endpoints

ACO was defined as the composite of all-cause death, new-onset arrhythmia, prosthetic valve failure, and repeat PVR. Time zero was defined as the date of PVR and the time to clinical outcomes was determined to be the first occurrence of ACO or the date of the last follow-up for those patients without an outcome. Early death was defined as death occurring ≤ 30 days after the initial operation or during the same hospitalization. Conversely, late death was defined as death occurring >30 days after the initial operation or after discharge. According to Khaled Alfakih's study (17), regardless of gender, normal RV volume was defined as RVEDVI ≤ 114 ml/m^2^, and RV normalization was defined as both RVEDVI ≤ 114 ml/m^2^ and RVEF ≥ 48%, by steady-state free precession imaging sequences. Cardiomegaly was defined as the cardiothoracic ratio ≥ 0.50 on posteroanterior chest X-ray.

### Statistical Analysis

Categorical variables were presented as frequencies and percentages. Continuous variables were presented as means ± standard deviation (SD) or medians with interquartile range (IQR). Comparisons between paired groups were performed using paired Student *t*-tests or the Wilcoxon signed-rank test as appropriate. Categorical variables were compared by χ^2^ and McNemar tests as appropriate. Bonferroni correction was applied when multiple comparisons were undertaken by dividing the original value of 0.05 by the number of analyses on the dependent variable (*k*). Survival estimates and the time to ACO were determined by the Kaplan–Meier analysis. Risk factors associated with ACO after PVR were identified by the Cox proportional hazards regression model. Linear regression analysis was performed to evaluate the association between two continuous variables. Statistical analysis was completed by SPSS Statistics Version 25 (IBM 16 Corporation, Armonk, New York) and R (version 3.1.2). A two-sided value of *p* < 0.05 was considered statistically significant.

## Results

### Patient Characteristics and Clinical Outcomes

The demographic characteristics of 42 patients are listed in Tables 1, 2. Surgical PVR was performed in 24 patients, and transcatheter PVR in 18 patients. Concomitant procedures during PVR included: tricuspid valvuloplasty in eight patients, right ventricular outflow tract muscle resection in three, residual ventricular septal defect closure in one patient, patent ductus arteriosus ligation in one patient, and major aortopulmonary collateral arteries occlusion in one patient. The mean cardiopulmonary bypass time was 190.9 ± 69.3 min, and the mean aortic cross-clamp time was 93.3 ± 34.6 min. The mean duration of hospital stay was 17 ± 8 days (Table 2).

**Table 1:** Demographics.

**Variables**	**Values**
Male	20 (48)
Age at TOF repair, years	2.0 (0.8-5.5)
Weight at TOF repair, kgs	9.3 (8.0-11.2)
**Previous palliative shunts**
Blalock-Taussig shunt	2 (5)
Modified Blalock-Taussig shunt	8 (19)
**Type of initial repair**
Transannular patch	32 (76)
Non-transannular patch	6 (14)
RV-to-PA conduit	4 (10)
Age at PVR, years	21.6 (15.4–24.8)
Time interval between TOF repair and PVR, years	16.4 (11.0–19.9)
Follow-up time, years	4.7 (4.2–5.0)
NYHA functional class
I	13 (31)
II	18 (43)
III	11 (26)
IV	0
TR grade
None	11 (26)
Trivial	4 (10)
Mild	18 (42)
Moderate	5 (12)
Severe	4 (10)

*Data are presented as n (%) or median (IQR). NYHA, New York Heart Association; PA, pulmonary artery; PVR, pulmonary valve replacement; RV, right ventricle; TR, tricuspid regurgitation; TOF, tetralogy of Fallot*.

**Table 2:** Perioperative characteristics and post-PVR outcomes.

**Variables**	**Values**
**Types of prosthetic pulmonary valve**
Surgical bioprosthetic	10 (24)
Homograft	14 (33)
Transcatheter bioprosthetic	18 (43)
Prosthetic pulmonary valve size, mm	26 (24–32)
**Concomitant procedures**
Tricuspid valve surgery	9 (21)
RVOT muscle resection	3 (7)
Residual VSD closure	1 (2)
PDA closure	1 (2)
MAPCA occlusion	1 (2)
CPB time, minutes	190.9 ± 69.3
ACC time, minutes	93.3 ± 34.6
Hospital stay, days	17 ± 8
**Post-PVR outcomes**
Re-intervention	4 (10)
New-onset arrhythmias	12 (29)
Prosthetic valve failure and dysfunction	4 (10)
Adverse clinical outcomes	16 (38)

*Data are presented as n (%), mean ± SD or median (IQR). ACC, aortic cross-clamp; CPB, cardiopulmonary bypass; MAPCA, major aortopulmonary collateral arteries; PDA, patent ductus arteriosus; PVR, pulmonary valve replacement; RVOT, right ventricular outflow tract; VSD, ventricular septal defect*.

The median duration of follow-up was 4.7 years (IQR, 4.2–5.0 years). About 74% of patients presented heart function in New York Heart Association Class I or II at baseline, and 95% maintained in New York Heart Association Class I or II by midterm follow-up after PVR (*p* < 0.001) (Table 3). Baseline QRS duration of 140 ± 31 ms on electrocardiogram (ECG) decreased with marginal statistical significance by midterm follow-up (140 ± 31ms vs. 111 ± 20 ms, *p* < 0.001). Cardiomegaly was documented in 40 (95%) patients preoperatively and reduced significantly by midterm follow-up after PVR (0.58 ± 0.05 vs. 0.49 ± 0.02, *p* < 0.001) (Table 3).

**Table 3:** Pre-PVR, early post-PVR, and midterm post-PVR variables of patients with rTOF.

**Variables**	**Pre-PVR**	**Early post-PVR**	**Midterm post-PVR**	**P value**		
				**Pre-PVR vs**.	**Early post-PVR vs**.	**Pre-PVR vs**.
				**Early post-PVR**	**Midterm post-PVR**	**Midterm post-PVR**
**CMR**
RVEDVI, mL/m^2^	155.2 ± 34.7	103.8 ± 31.2	95.1 ± 28.6	<0.001	<0.001	<0.001
RVESVI, mL/m^2^	102.9 ± 28.5	65.4 ± 28.2	57.7 ± 23.4	<0.001	<0.001	<0.001
RVEF, %	35.1 ± 8.8	37.9 ± 10.1	41.2 ± 8.7	0.06	<0.001	<0.001
PR fraction, %	38.1 ± 8.2	5.1 ± 3.6	4.7 ± 3.7	<0.001	0.74	<0.001
LVEDVI, mL/m^2^	69.2 ± 14.1	77.6 ± 18.1	77.5 ± 14.9	<0.001	0.52	0.001
LVESVI, mL/m^2^	36.2 ± 9.9	36.2 ± 11.3	37.8 ± 11.1	0.93	0.30	0.99
LVEF, %	48.1 ± 7.8	53.8 ± 6.6	56.1 ± 6.4	<0.001	<0.001	<0.001
**Echocardiography**
RVAPD, mm	33.8 ± 8.3	29.1 ± 7.7	28.5 ± 6.0	<0.001	0.42	<0.001
RVSP, mmHg	17.5 ± 14.1	14.6 ± 8.2	13.0 ± 6.7	0.41	0.14	0.17
QRS duration, ms	140 ± 31	134 ± 30	111 ± 20	0.039	<0.001	<0.001
Cardiothoracic ratio	0.58 ± 0.05	0.51 ± 0.05	0.49 ± 0.02	<0.001	0.037	<0.001
NYHA functional class I/II/III/IV	13/18/11/0	32/9/1/0	39/2/1/0			<0.001
**Grade of TR**, ***n*** **(%)**
None/trivial/mild	33 (79)		40 (95)			
Moderate/severe	9 (21)		2 (5)			0.021

*Data are presented as n (%) or mean ± SD. CMR, cardiac magnetic resonance; LVEDVI, left ventricular end-diastolic volume index; LVESVI, left ventricular end-systolic volume index; LVEF, left ventricular ejection fraction; NYHA, New York Heart Association; PVR, pulmonary valve replacement; PR, pulmonary regurgitation; RVEDVI, right ventricular end-diastolic volume index; RVESVI, right ventricular end-systolic volume index; RVEF, right ventricular ejection fraction; RVAPD, right ventricular anteroposterior diameter; RVSP, right ventricular systolic pressure; TR, tricuspid regurgitation*.

**Figure 1:**
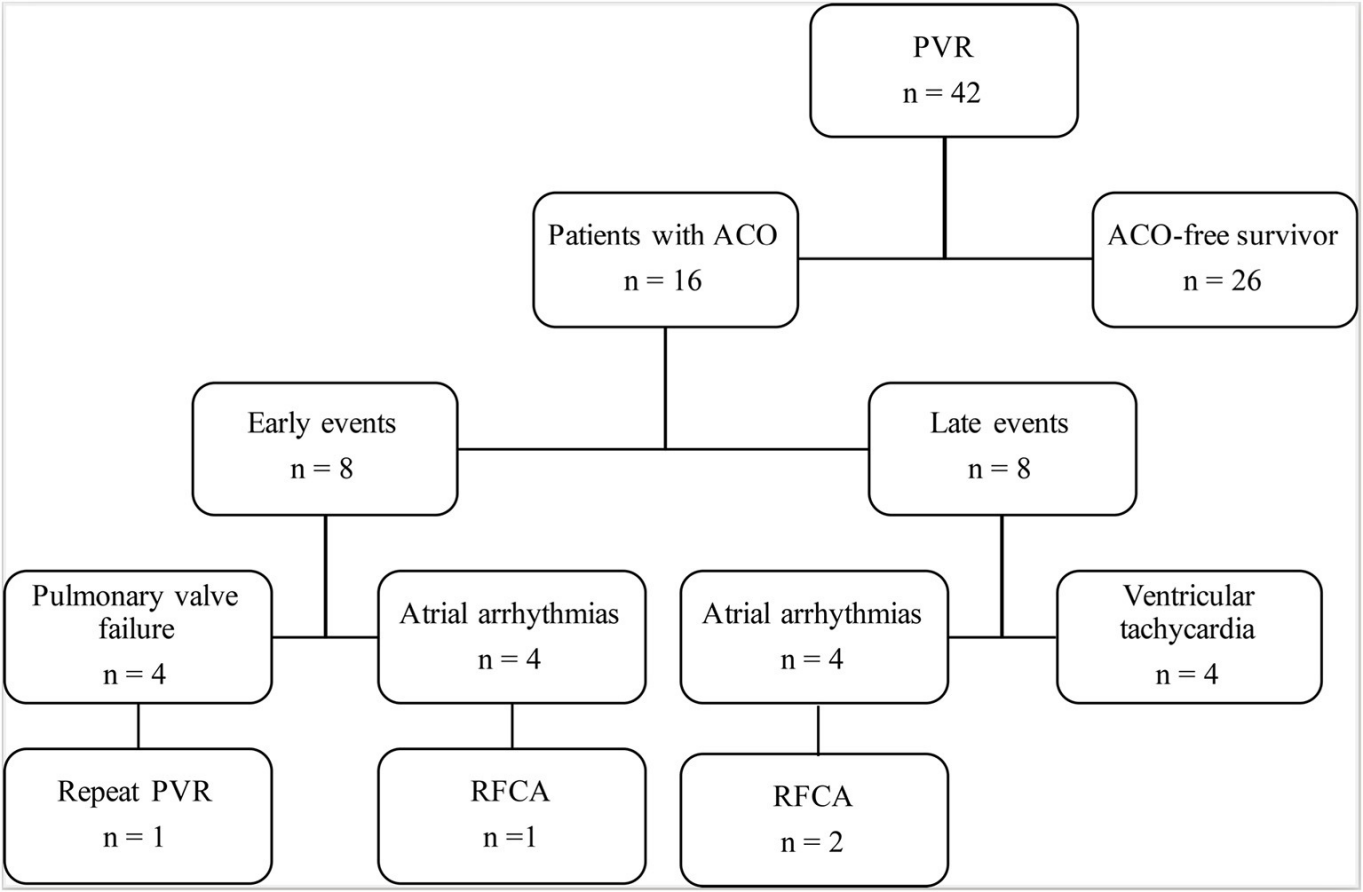
Follow-up and outcomes after PVR. ACO, adverse clinical outcomes; PVR, pulmonary valve replacement; RHCA, radiofrequency catheter ablation.

**Figure 2:**
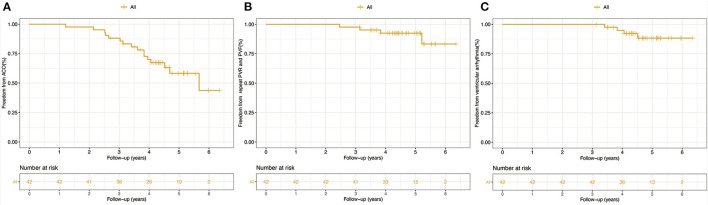
Kaplan-Meier analysis for clinical outcomes. **(A)** Freedom from ACO. **(B)** Freedom from repeat PVR and PVF. **(C)** Freedom from new-onset arrhythmia. ACO, adverse clinical outcomes; PVR, pulmonary valve replacement; PVF, pulmonary valve failure.

There was no early or late death in this study. ACO occurred in 16 (38%) patients: prosthetic valve failure in four patients, and new-onset arrhythmia in 12 (Figure 1). Freedom from ACO at 3 and 5 years was 88.1 ± 5% and 58.2 ± 9%, respectively (Figure 2A). One of four patients with developed prosthetic valve failure accepted a repeat PVR in the third year after the initial PVR. Freedom from repeat PVR and prosthetic valve failure at 3 and 5 years was 97.6 ± 2% and 92.5 ± 4%, respectively (Figure 2B). For patients with new-onset arrhythmia (ventricular arrhythmia in four patients, and sustained atrial arrhythmia in eight patients), three patients with atrial flutter were indicated to necessary radiofrequency catheter ablation treatment, and four patients developed non-sustained ventricular tachycardia but without requiring intervention. Freedom from new-onset ventricular arrhythmia at 3 and 5 years was 97.6 ± 2.4% and 88.3 ± 5.7%, respectively (Figure 2C).

### Cardiovascular Magnetic Resonance

The early postoperative CMR was performed at a median time of 1.0 year (IQR, 0.5–1.7 years) and the midterm postoperative CMR at a median time of 4.2 years (IQR, 3.5–4.8 years) after PVR. Massive RV dilation (RVEDVI ≥ 200 ml/m^2^) was only detected in five patients on preoperative CMR. Compared with the baseline, there was a 33% reduction in RVEDVI by the early post-PVR period (155.2 ± 34.7 vs. 103.8 ± 31.2 ml/m^2^, *p* < 0.001), which decreased further to 39% by the midterm follow-up (103.8 ± 31.2 vs. 95.1 ± 28.6 ml/m^2^, *p* < 0.001). RVESVI promptly decreased by early post-PVR period to 36% (102.9 ± 28.5 vs. 65.4 ± 28.2 mL/m^2^, *p* < 0.001) and decreased further by the midterm follow-up to 44% lower than the baseline (102.9 ± 28.5 vs. 57.7 ± 23.4 ml/m^2^, *p* < 0.001) (Table 3). Compared with the baseline, RVEF increased by 17% at midterm follow-up (35.1 ± 8.8 vs. 41.2 ± 8.7 %, *p* < 0.001). Normal RV volume was noted in 35 patients, and RV normalization occurred in 21 (50%) patients by midterm follow-up. Left ventricular end-diastolic volume index increased by 12% early after PVR (69.2 ± 14.1 vs. 77.6 ± 18.1 ml/m^2^, *p* < 0.001) and sustained at midterm follow-up. Left ventricular end-systolic volume index (LVESVI) only increased by 4% at midterm follow-up (36.2 ± 9.9 vs. 37.8 ± 11.1 ml/m^2^, *p*=0.99). Left ventricular ejection fraction (LVEF) increased by 12% early after PVR (48.1 ± 7.8 vs. 53.8 ± 6.6 %, *p* < 0.001) and continued the improvement of 4% at midterm follow-up (53.8 ± 6.6 vs. 56.1 ± 6.4 %, *p* < 0.001).

**Figure 3:**
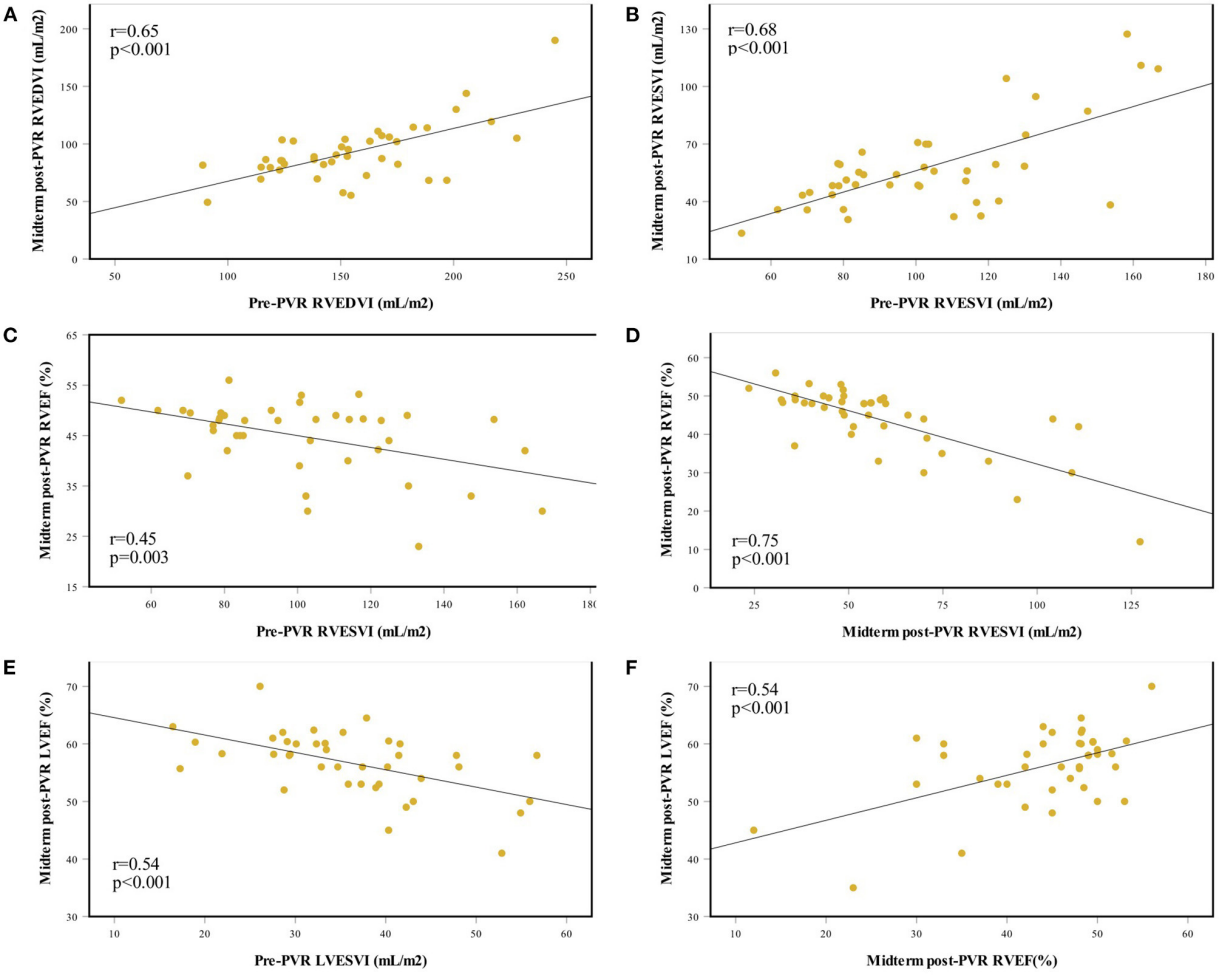
Associations between pre-PVR and midterm post-PVR CMR parameters. **(A)** Association between pre-PVR and midterm post-PVR RVEDVI. **(B)** Association between pre-PVR and midterm post-PVR RVESVI. **(C)** Association between pre-PVR RVESVI and midterm post-PVR RVEF. **(D)** Association between midterm post-PVR RVESVI and RVEF. **(E)** Association between pre-PVR LVESVI and midterm post-PVR LVEF. **(F)** Association between midterm post-PVR RVEF and LVEF. CMR, cardiac magnetic resonance; LVESVI, left ventricular end-diastolic volume index; LVEF, left ventricular ejection fraction; PVR, pulmonary valve replacement; RVEDVI, right ventricular end-diastolic volume index; RVESVI, right ventricular end-systolic volume index; RVEF, right ventricular ejection fraction.

Figure 3 demonstrates the correlations between pre- and midterm post-PVR CMR parameters. Pre-PVR RV volumes were associated with midterm post-PVR RV volumes (RVEDVI, *r* = 0.65, *p* < 0.001; RVESVI, *r* = 0.68, *p* < 0.001). Lower midterm post-PVR RVEF was associated with increasing pre-PVR and midterm post-PVR RVESVI. Lower midterm post-PVR LVEF was associated with lower midterm post-PVR RVEF (*r* = 0.54, *p* < 0.001).

### Factors Associated With ACO After PVR

Age at TOF repair, age at PVR, transannular repair, New York Heart Association (NYHA) function class III or IV, moderate or severe TR, CPB time, ACC time, and pre-PVR examination parameter were included in the univariable analysis. Among parameters of pre-PVR examination, larger RVEDVI [hazard ratio (HR) = 1.02, 95% confidence interval (CI) 1.00–1.03; *p* = 0.038], larger RVESVI (H*R* = 1.02, 95% CI 1.00–1.04; *p* = 0.035), lower RVEF (H*R* = 1.03, 95% CI 1.01–1.12; *p* = 0.042), lower LVESVI (H*R* = 1.05, 95% CI 1.00–1.10; *p* = 0.030), and lower LVEF (H*R* = 1.02, 95% CI 1.00–1.14; *p* = 0.026) were associated with ACO in the univariable analysis. In the multivariable analysis, however, lower preoperative LVESVI was identified as a sole independent risk factor for ACO (Table 4).

**Table 4:** Risk factors associated with adverse clinical outcomes after PVR.

**Variables**	**Univariable analysis**			**Multivariable analysis**		
	**HR**	**95% CI**	***P*** **-value**	**HR**	**95% CI**	***P*** **-value**
**Patient and surgical characteristics**
Age at TOF repair, years	0.95	0.89–1.02	0.18			
Age at PVR, years	0.98	0.94–1.03	0.51			
Transannular repair	0.36	0.09–1.40	0.14			
NYHA functional class III or IV	1.69	0.84-3.39	0.13			
Moderate or severe TR	1.36	0.95–1.96	0.08			
CPB time, minutes	1.01	0.99–1.02	0.22			
ACC time, minutes	1.01	0.99–1.04	0.19			
**Pre-PVR examination parameters**
RVEDVI, mL/m^2^	1.02	1.00–1.03	0.038			
RVESVI, mL/m^2^	1.02	1.00–1.04	0.035			
RVEF, %	1.03	1.01–1.12	0.042	0.99	0.95–1.01	0.32
PR fraction, %	0.97	0.91–1.04	0.49			
LVEDVI, mL/m^2^	1.02	0.99–1.05	0.14			
LVESVI, mL/m^2^	1.05	1.00–1.10	0.030	1.05	1.00–1.10	0.034
LVEF, %	1.02	1.00–1.14	0.026			
QRS duration, ms	1.01	0.99–1.03	0.18			

*ACC, aortic cross-clamp; CPB, cardiopulmonary bypass; LVEDVI, left ventricular end-diastolic volume index; LVESVI, left ventricular end-systolic volume index; LVEF, left ventricular ejection fraction; NYHA, New York Heart Association; PVR, pulmonary valve replacement; PR, pulmonary regurgitation; RVEDVI, right ventricular end-diastolic volume index; RVESVI, right ventricular end-systolic volume index; RVEF, right ventricular ejection fraction; TOF, tetralogy of Fallot; TR, tricuspid regurgitation*.

## Discussion

Our study demonstrated an acceptable midterm outcome of PVR with reversible RV remodeling in patients with rTOF. Freedom from ACO at 3 and 5 years was 88 and 58%, respectively. Notably, we observed a remarkable reduction of RV volumes on CMR through a follow-up of 4.7 years, accompanied by a significant improvement in RV and LV function.

### Midterm Outcomes of PVR

With the increasing emphasis on cut-off values of preoperative RV volume in determining the optimal timing of PVR, a proactive approach is predominating the surgical strategy for patients with rTOF (18–21). Therein, the improved event-free survival rate was encouraging. Cheung et al. (7) reported low operative mortality of 1% to 4% for PVR, and our study has confirmed this finding. Also consistent with previous studies (2, 10, 22–25), we showed a favorable midterm ACO-free survival of 88.1% at 3 years. Impaired LV function (LVEF <50%) and large RV volumes (RVEDVI > 150 ml/m^2^) were documented in those four patients with sustained ventricular tachycardia before PVR. Our results might correspond with the finding of earlier studies showing that PVR did not reduce the occurrence of ventricular arrhythmias, particularly for those with high preoperative RV volumes and LV impairment (12, 26). Nevertheless, careful surveillance and routine ECGs examinations during follow-up are warranted for adult patients with rTOF.

### RV and LV Reverse Remodeling

On CMR, we demonstrated the marked reduction of RV volumes and improvement of biventricular function during the follow-up time of 4.7 years. Hallbergson et al. (27) reported similar results of early reduction in RVEDVI and RVESVI. In accordance with their findings, a subsequent decline of RV volume might not occur after PVR, and even a gradual rebound of RV volume toward preoperative values would take place, for which the late deterioration of implanted pulmonary valve could be to blame. On the contrary, the continued improvements of RV size and function were found by midterm follow-ups in this series. These different changes might correlate with the decreased occurrence of late prosthetic valve failure, given favorable freedom from repeat PVR and pulmonary valve failure and dysfunction at 3 and 5 years (97.6 and 92.5%, respectively). Meanwhile, Heng et al. (14) revealed that rapid reduction of RV volumes after PVR might be followed by time-dependent biological remodeling by midterm follow-up. Our data supported this finding. As the ongoing improvement of RV function, however, seemed to appear a “slow-down” reduction of both RVEDVI and RVESVI from the early post-PVR period to midterm follow-up. Considering the close correlation between lower RVEF and higher RVESVI, our findings indicated that post-PVR RV normalization might occur in a time-dependent sequence from ventricular dilation to remodeling.

Of note, although the majority (83%) of patients regained normal RV volume, RV normalization merely occurred in half of the study population. This might imply that too much emphasis on preoperatively RVEDVI would be insufficient for predicting RV normalization after PVR. After all, achieving RV normalization is important for the improvement of long-term outcomes (4, 23). Meanwhile, we observed a close correlation between the progressive reduction of RVESVI and continued improvement of RVEF, justifying the potential use of RVESVI in predicting the intrinsic RV normalization. Additionally, larger RVESVI and lower RVEF were identified to be associated with ACO in the univariable analysis. In summary, our findings verified the diagnostic combination of preoperative RVESVI and RVEF in determining the optimal timing of PVR, which calls into question the current focus on CMR-based pre-PVR threshold values of RVEDVI that predicts RV normalization.

Previous studies have suggested the association between reverse RV remodeling and improvement of LV function (9, 28, 29). In our study, we also found that higher LVEF was associated with increasing RVEF by midterm follow-up. With pulmonary valve competency restored by PVR, normalized RV cardiac output leads to increased LV filling and volumes, and resultant increased LVEF?that is, the positive interaction between RV and LV. This might explain the symptomatic benefits of our patients, wherein the majority (95%) of them had regained normal exertion capacity in NYHA class I or II by midterm follow-up.

### Predictors of ACO After PVR

In this study, predictors of ACO including larger preoperative RV volume, depressed RV function, and lower LV function were identified in the univariate analysis, which was consistent with reported findings of previous studies (13, 18, 30, 31). Interestingly, age at PVR for predicting adverse outcomes is still sparking debate. Jang et al. (32) found that early PVR might decrease the durability of implanted valves. Conversely, Lee et al. (30) reported that patients with older age at TOF repair and older age at PVR were at increased risk for ACO. These two factors, however, were not found to be associated with ACO in our study. The median time interval between TOF repair and PVR in this cohort was 16.4 years, which was in line with the previously suggested time interval of 20 years after TOF repair (33). Similar to previous studies describing the prognostic value of LV function in rTOF (14, 31), lower LVESVI was identified as an independent risk factor for ACO.

## Limitations

By design, this cohort is restricted to patients who had undergone three complete CMR. Patients with incomplete CMR or contraindications to CMR were excluded, which certainly reduce the population size. Additionally, since the majority of variables were time-dependent, the time interval between PVR and postoperative CMR study is another significant limitation of our study. A long-term follow-up on the continuous benefits of PVR is warranted.

## Conclusions

The midterm outcome of PVR in patients with repaired TOF was favorable with the improvement of biventricular function. Preoperative LVESVI on CMR was the independent predictor for adverse clinical outcomes after PVR.

## Data Availability Statement

The raw data supporting the conclusions of this article will be made available by the authors, without undue reservation.

## Ethics Statement

The studies involving human participants were reviewed and approved by the Ethics Committee of Fuwai Hospital. Written informed consent to participate in this study was provided by the participants' legal guardian/next of kin.

## Author Contributions

FH, ZF, and SL conceived and designed the research. JY, KM, and SZ performed the research. KY and SL performed the surgery. ML analyzed the cardiovascular magnetic resonance data. FH and ZF analyzed the data and wrote the article. All authors listed have made a substantial, direct, and intellectual contribution to the work and approved it for publication.

## Conflict of Interest

The authors declare that the research was conducted in the absence of any commercial or financial relationships that could be construed as a potential conflict of interest.

## Publisher's Note

All claims expressed in this article are solely those of the authors and do not necessarily represent those of their affiliated organizations, or those of the publisher, the editors and the reviewers. Any product that may be evaluated in this article, or claim that may be made by its manufacturer, is not guaranteed or endorsed by the publisher.
