# Supplementary material for: Pulmonary Valve Replacement in Repaired Tetralogy of Fallot: Midterm Impact on Biventricular Response and Adverse Clinical Outcomes
Source: Front Pediatr. 2022 May 6;10:864455. doi: 10.3389/fped.2022.864455 (PMC9120843; doi:10.3389/fped.2022.864455)
Supplement: Supplementary file 1 [file Data_Sheet_1.doc]

**Supplementary materials**

**Table 1. Demographics before exclusion**

| Variables |  |  |  | Values |
| --- | --- | --- | --- | --- |
| Male |  |  |  | 23 (51) |
| Age at TOF repair, years |  |  |  | 2.0 (0.8-5.5) |
| Weight at TOF repair, kgs |  |  |  | 9.0 (8.0-11.2) |
| Previous palliative shunts |  |  |  |  |
| Blalock-Taussig shunt |  |  |  | 3 (7) |
| Modified Blalock-Taussig shunt |  |  |  | 10 (22) |
| Type of initial repair |  |  |  |  |
| Transannular patch |  |  |  | 35 (76) |
| Non-transannular patch |  |  |  | 6 (14) |
| RV-to-PA conduit |  |  |  | 4 (10) |
| Age at PVR, years |  |  |  | 22.5 (15.4-24.8) |
| Time interval between TOF repair and PVR, years |  |  |  | 16.4 (11.2-19.9) |
| Follow-up time, years |  |  |  | 4.7 (4.2-5.1) |
| NYHA functional class |  |  |  |  |
| I |  |  |  | 12 (27) |
| II |  |  |  | 19 (42) |
| III |  |  |  | 11 (26) |
| IV |  |  |  | 0 |
| TR grade |  |  |  |  |
| None |  |  |  | 12 (27) |
| Trivial |  |  |  | 6 (14) |
| Mild |  |  |  | 18 (42) |
| Moderate |  |  |  | 5 (12) |
| Severe |  |  |  | 4 (10) |

Data are presented as n (%) or median (IQR). NYHA, New York Heart Association; PA, pulmonary artery; PVR, pulmonary valve replacement; RV, right ventricle; TR, tricuspid regurgitation; TOF, tetralogy of Fallot.

Table 2. Surgical characteristics and post-PVR outcomes before exclusion

| Variables |  |  |  | Values |
| --- | --- | --- | --- | --- |
| Type of prosthetic pulmonary valve |  |  |  |  |
| Surgical bioprosthetic |  |  |  | 14 (31) |
| Homograft |  |  |  | 15 (36) |
| Transcatheter |  |  |  | 16 (38) |
| Prosthetic pulmonary valve size, mm |  |  |  | 26 (25-30) |
| Concomitant procedures |  |  |  |  |
| Tricuspid valve surgery |  |  |  | 9 (21) |
| RVOT muscle resection |  |  |  | 3 (7) |
| Residual VSD closure |  |  |  | 1 (2) |
| PDA closure |  |  |  | 1 (2) |
| MAPCA occlusion |  |  |  | 1 (2) |
| CPB time, minutes |  |  |  | 190.5 ± 69.3 |
| ACC time, minutes |  |  |  | 93.3 ± 34.6 |
| Hospital stay, days |  |  |  | 16 ± 8 |
| Post-PVR outcomes |  |  |  |  |
| Re-intervention |  |  |  | 4 (10) |
| New-onset arrhythmias |  |  |  | 12 (29) |
| Prosthetic valve failure and dysfunction |  |  |  | 4 (10) |
| Adverse clinical outcomes |  |  |  | 16 (38) |

Data are presented as n (%), mean ± SD or median (IQR). ACC, aortic cross-clamp; CPB, cardiopulmonary bypass; MAPCA, major aortopulmonary collateral arteries; PDA, patent ductus arteriosus; PVR, pulmonary valve replacement; RVOT, right ventricular outflow tract; VSD, ventricular septal defect.
